# Assessment of a High Sensitivity Method for Identification of *IDH1* R132x Mutations in Tumors and Plasma of Intrahepatic Cholangiocarcinoma Patients

**DOI:** 10.3390/cancers11040454

**Published:** 2019-03-30

**Authors:** Caterina Peraldo-Neia, Maria Scatolini, Enrico Grosso, Pasquale Lombardi, Roberto Filippi, Chiara Raggi, Caterina Marchiò, Giuliana Cavalloni, Massimo Aglietta, Francesco Leone

**Affiliations:** 1 Cancer Genomics Laboratory, Fondazione Edo ed Elvo Tempia, Via Malta 3, 13900 Biella, Italy; 2 Laboratory of Molecular Oncology, Fondazione Edo ed Elvo Tempia, Via dei Ponderanesi 2, 13875 Ponderano, Biella, Italy; maria.scatolini@fondazionetempia.org (M.S.); enrico.grosso@fondazionetempia.org (E.G.); 3 Department of Oncology, University of Turin, 10100 Torino, Italy; pasquale.lombardi@unito.it (P.L.); roberto.filippi@unito.it (R.F.); massimo.aglietta@ircc.it (M.A.); francesco.leone@unito.it (F.L.); 4 Department of Experimental and Clinical Medicine, University of Firenze, 50100 Firenze, Italy; chiara.raggi@unifi.it; 5 Center for Autoimmune Liver Diseases, Humanitas Clinical and Research Center, 20089 Rozzano, Italy; 6 Department of Medical Sciences, University of Turin, 10100 Torino, Italy; caterina.marchio@ircc.it; 7 Pathology Unit, Candiolo Cancer Institute-FPO-IRCCS, Candiolo, 10060 Torino, Italy; 8 Division of Medical Oncology, Candiolo Cancer Institute, FPO-IRCCS, Candiolo, 10060 Torino, Italy; giuliana.cavalloni@ircc.it

**Keywords:** IDH1 mutation, intrahepatic cholangiocarcinoma, qPCR, liquid biopsy, biomarker

## Abstract

Hotspot codon 132 mutations (R132x*IDH1*m) are frequent in intrahepatic cholangiocarcinoma (ICC), are druggable by anti-*IDH1*m agents, and could represent a marker of disease progression. Developing an assay to identify R132x*IDH1*m would provide a useful tool to select patients benefitting from targeted treatments. We tested a quantitative real-time allele-specific polymerase chain reaction (qPCR)-based method to detect the main R132x*IDH1*m in an Italian ICC series (*n* = 61) of formalin-fixed paraffin-embedded (FFPE) samples, and on circulating-free DNA samples. The outcomes were compared with nested PCR/Sanger sequencing. Reconstitution experiments of plasmids harboring the different R132x*IDH1*m mixed with wild-type (WT) DNA demonstrated that qPCR is able to detect at least 2% of all mutated allele. High efficiency was also observed on patient-derived mutated DNA mixed with WT DNA (up to 10% and 0.3 ng of mutated template); qPCR detected 16.4% of mutated samples (one R132G, three R132C and six R132L) while nested PCR/Sanger sequencing only 8.2% (four R132L and one R132G). In a single patient with an R132C-mutated tumor, qPCR was also performed on plasma samples collected at four time-points, observing an increase correlating with disease progression. In conclusion, we developed a qPCR assay which could represent a fast, inexpensive and sensitive tool both for detection of R132x*IDH1*m in ICC samples and monitoring disease progression from liquid biopsy.

## 1. Introduction

Intrahepatic cholangiocarcinoma (ICC) is a malignancy arising from the epithelial cells of the intrahepatic bile duct branches of the second-order or beyond. The incidence of ICC accounts for 10–20% of all cholangiocarcinomas (CCA) and 3% of gastrointestinal carcinomas [1,2]. The etiological spectrum varies with the geographical region [3]. The median survival time in ICC patients is as low as 2–6 months if only palliative treatment is administered. The 5 year survival rate after surgical resection still lies around 30–35% [4]. Although surgery is the main treatment option for early ICC, only approximately one-third of ICC patients undergo surgical resection. Survival time in the advanced setting exceeds 5 years in less than 10% of patients, regardless of the systemic treatment employed [5]. Since chemotherapy approaches have provided poor benefits in terms of survival, deep molecular studies have been conducted in the last years, with the aim of identifying molecular drivers as well as potential targets for tailored therapies. In particular, the mutational status of the main ICC cancer related genes was analyzed in different cohorts of patients. Globally, among the most frequently mutated genes, literature data reported *KRAS*, *BRAF*, *TP53*, *ARID1A*, *MLL3*, *ROBO2*, *RNF43*, *PEG3*, *GNAS*, *IDH1/2*, *BAP1*, *FGFR* and *ROS* fusions [6,7,8,9,10,11], suggesting potential targets for therapy. Diverse factors, encompassing ethnicity, risk factors and hepatic localization could influence the mutational spectrum of ICC. In fact, Ong and collaborators analyzed a cohort of liver fluke cholangiocarcinomas, evidencing a different mutational landscape than in non-liver fluke infected patients [8]. The incidence in *BRAF* and *KRAS* mutations is higher in Asian than European countries. In a recent study by Farshidfar and collaborators, a deep molecular investigation was performed on 38 CCA samples, mainly ICC, analyzed for DNA and RNA-seq and crossed with data obtained from The Cancer Genome Atlas (TCGA) and other datasets—4 different clusters were identified on the basis of the mutation and aberration profiles [12]. A particular subgroup of ICC resulted enriched for *IDH1* mutation (IDH1m) and is characterized by DNA hypermethylation, which influenced the gene expression and the cell differentiation [13]. The prevalence of the codon 132 hotspot (R132x) IDH1m in ICC is estimated to be 10–30% [14]. In a recent work, we analyzed the transcriptomic and mutational profiles of paired primary and recurrent ICCs—we found that 5 out of 15 patients (33%) harbored an *IDH1* R132x mutation; of them, 4 displayed a naïve mutation only in the recurrent counterpart, suggesting that IDH1m could be a marker of progression in ICC [15]. The prognostic role of IDH1m is still controversial, with reports of better survival outcomes, of no association with survival at all [16,17,18,19], and even of a detrimental effect on prognosis [20]. Due to its role as oncogene, the IDH1m has recently become an attractive target for therapies, and some clinical trials are ongoing using specific IDH1m inhibitors, in particular, in gliomas, hematologic malignancies and CCAs (NCT02074839; NCT02989857; NCT03127735). Here, we evaluated the incidence of mutations in the exon 4 of *IDH1*, focusing on the *IDH1* R132x mutation, in an Italian series of 61 primary ICCs. We set up a quantitative real-time allele-specific polymerase chain reaction (qPCR) assay to detect with high efficiency, comparable with next generation sequencing (NGS) data [21], the specific IDH1m both in tissue and in plasma samples. In particular, the reliability and sensitivity of the technique on tissues may extend the number of patients in whom the IDH1m is detectable at an early stage and who can benefit from targeted therapy. Moreover, the efficiency of the qPCR in plasma samples could provide a non-invasive tool to monitor the progression of the disease. We also compared the sensitivity of qPCR with Sanger sequencing, the conventional and cheapest method to detect gene mutations, frequently used in routine molecular diagnostics, but characterized by lower sensitivity.

## 2. Results

### 2.1. Sensitivity of IDH1 R132x qPCR

We developed a qPCR assay able to identify the main *IDH1*R132x mutations (R132C, R132G, R132H, R132L, R132S) in formalin-fixed paraffin-embedded (FFPE) samples and cfDNA.

Standard curves were performed on mutated/wild-type (WT) positive controls (from 3 × 10^3^ to 3 *IDH1* copies) showing a mean efficiency (E) of 98.9%. Specific efficiency for each *IDH1* mutation and for the WT allele are summarized in Table 1. The efficiency is variable according to the mutation analyzed.

In order to simulate different percentages of mutated allele in the sample (from 8% to 2%; from ~2500 to 500 *IDH1* gene copies), plasmids harboring the different *IDH1* mutants were mixed with *IDH1* WT DNA—as shown in Table 2, qPCR is able to detect up to 2% of mutated allele, except for the R132H mutant.

Then, we tested the efficiency of the method in experiments in which DNA derived from three patients with *IDH1* mutations (two samples with R132L and one with R132G mutations, respectively) was mixed with *IDH1* WT DNA at different proportions (100%, 50%, 10%). In this case, we were able to detect the mutations up to 10% diluted mutated samples (~5% mutated allele), but only in two out of three samples, as shown in Table 3.

**Figure 1:**
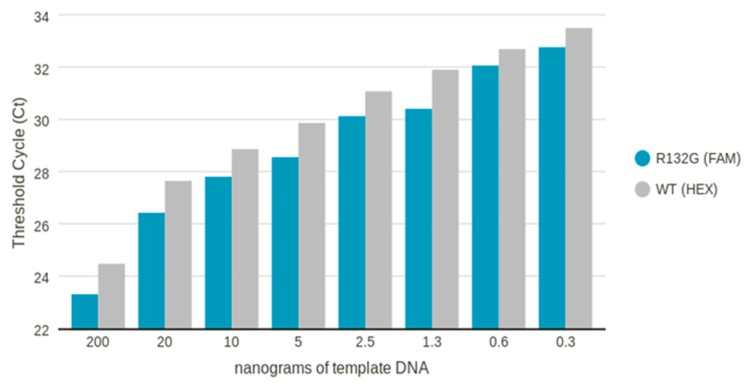
Mean Ct obtained on diluted genomic DNA isolated from R132G mutated sample.

We also performed a DNA dilution experiment in which the R132G mutated sample was diluted from 200 to 0.3 ng and we demonstrated that qPCR is able to detect up to 0.3 ng of mutated template, as shown in Figure 1.

### 2.2. Comparison between Quantitative PCR (qPCR) and Nested PCR for the Identification of IDH1R132x Mutations

A series of 61 ICC samples was analyzed to identify the frequency of *IDH1* exon 4 mutations, using both qPCR and nested PCR followed by Sanger sequencing.

**Figure 2:**
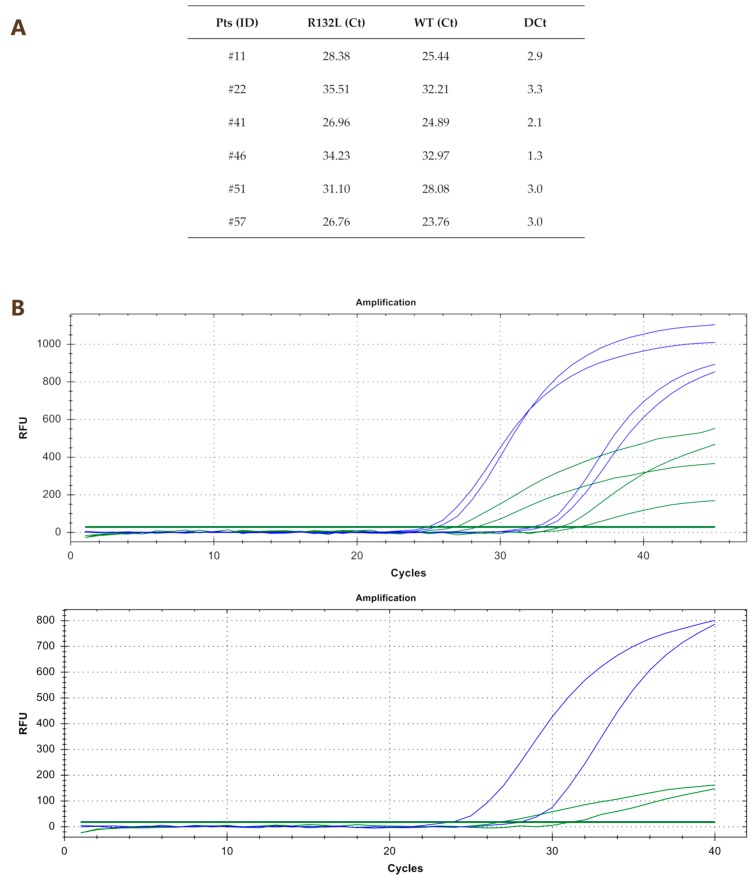
Ct values (panel **A**) and amplification curves (panel **B**) of qPCR of R132L mutated samples. RFU indicates relative fluorescent units.

**Figure 3:**
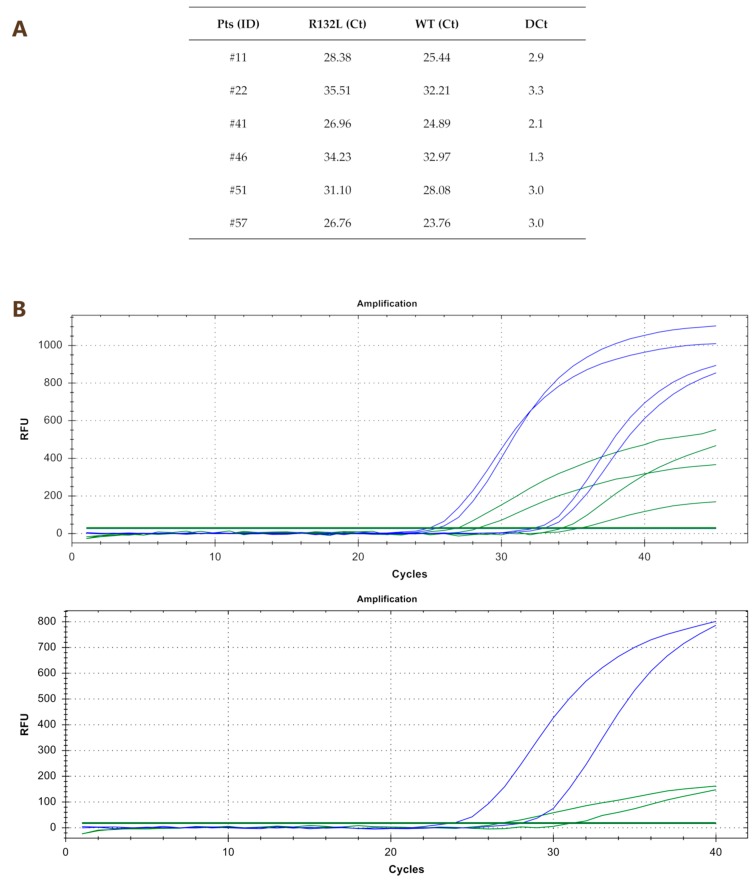
Ct values and amplification curves of qPCR of R132C (panel **A**) and of R132G (panel **B**) mutated samples. RFU indicates relative fluorescent units.

By using qPCR with specific probes for each mutation (R132C/G/H/L/S), we detected 10 out of 61 mutated samples (16.4%). In particular, six patients displayed the R132L mutation (#11, #22, #41, #46, #51 and #57), three patients the R132C mutation (#15, #21 and #45) and one patient (#61) the R132G mutation. Ct values and amplification curves of qPCR of R132L and R132C/G mutated samples are reported in Figure 2 and Figure 3, respectively.

**Figure 4:**
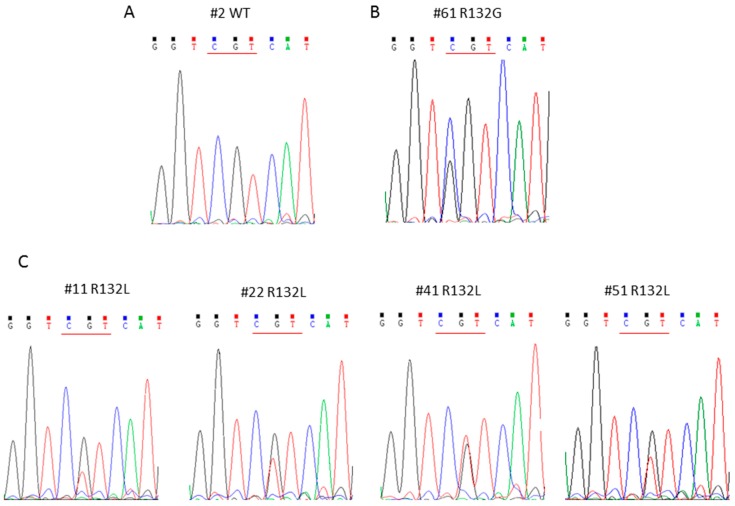
Representative electropherograms of *IDH1* WT (**A**), R132G (**B**) and R132L (**C**) mutated samples.

The same cohort was analyzed by nested PCR followed by Sanger sequencing. With this technique, we were able to detect the mutations only in 5 out of 61 patients (8.2%); in particular, 4 patients displayed the R132L (#11, #22, #41 and #51), as shown in Figure 4C, and one the R132G mutation (#61), as shown in Figure 4B. The WT sequence is shown in Figure 4A.

**Figure 5:**
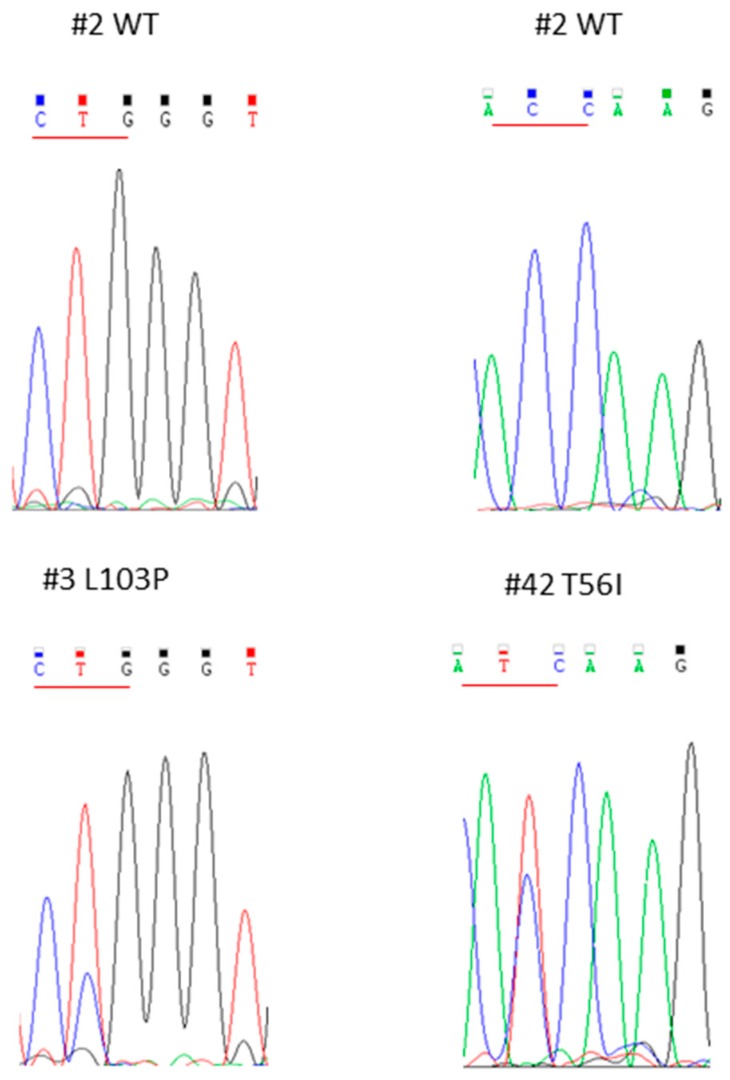
Representative electropherograms of two novel mutations found in *IDH1* exon 4.

Moreover, with Sanger sequencing we found also two novel, heterozygous point-missense mutations within *IDH1* exon 4, as shown in Figure 5; in particular, the mutation at codon 102 resulted in the substitution of lysine with proline in patient #3, whereas the mutation at codon 56 resulted in the substitution of threonine with isoleucine in patient #42.

Table 4 summarizes the mutations detected with the two techniques.

### 2.3. cfIDH1m R132x is a Potential Prognostic Marker in ICC in Plasma Samples

**Figure 6:**
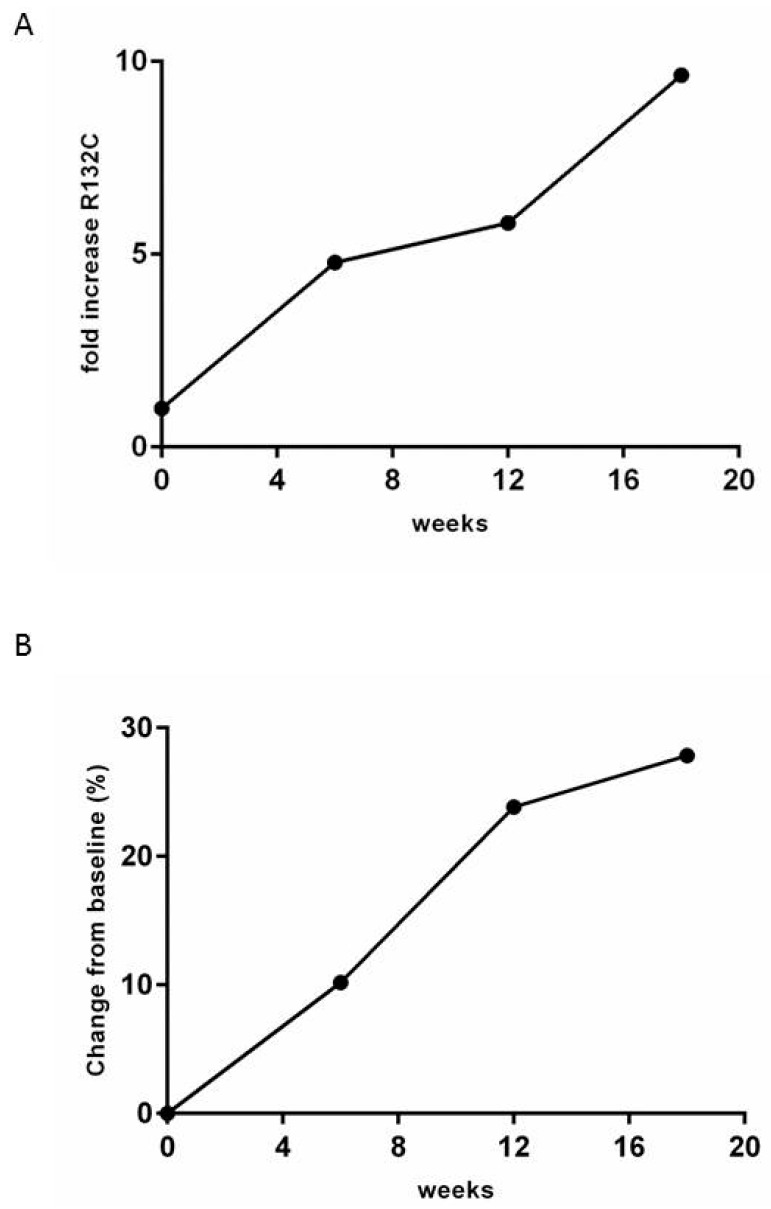
(**A**) Fold increase values of mutated allele in seriate longitudinal plasma samples collected every 6 weeks. The value is obtained normalizing on T0. (**B**) Trend of tumor diameter increase of selected lesions evaluated at the baseline, and every 6 weeks by CT scan. These time-points corresponded to the plasma collection used for the evaluation of IDH1m (T0 and every 6 weeks).

For one patient, both the tumor biopsy and plasma samples (obtained at four different time points, at the beginning of systemic treatment and every six weeks afterwards) were available. Both Sanger sequencing and qPCR detected the *IDH1*m R132C in tissue biopsy. The liquid biopsy analysis for this specific mutation performed on cfDNA revealed a progressive decrease of Ct, as it indicates an increase of the mutated copies, from the first blood sampling to the latter, as shown in Figure 6A and Figures S1–S3, suggesting that the mutated allele load is directly correlated with the disease progression evaluated by CT scan, as shown in Figure 6B.

## 3. Discussion

In this work, we analyzed the incidence of *IDH1* mutation in the hotspot codon 132 in an Italian series of FFPE ICC samples and assessed two different techniques for detection of the mutation, the allele specific qPCR, and the classical Sanger sequencing. We reported that 16.4% of patients harbored one of the *IDH1*R132x mutations, similar to other studies [17,22,23,24,25]; however, only 5 out of 10 mutations were also detected also by Sanger sequencing (8.2%). We found that six samples carried the R132L mutation, three samples the R132C and one the R132G; these mutations are already described in ICC [25,26]. The absence of the R132H mutation, typical of glioblastoma, confirmed the already reported data which described that ICCs have a different mutational pattern [27]. The difference in sensitivity between the two techniques indicates that nested PCR followed by Sanger sequencing is able to detect mutations only if the biological material is well preserved and probably if the mutated clone is overrepresented; although this method is the cheapest one, data suggest that it is not efficient to screen FFPE samples for mutations. We assessed the sensitivity of Sanger sequencing at 20 ng (Figure S4); this data could also be influenced by scarce preservation of biological materials (i.e., DNA fragmentation, formaldehyde-damaged DNA by formation of DNA-protein crosslinks, and DNA breaks) which could impair the nested PCR due to longer primers compared to qPCR.

We performed different tests to assess the sensitivity of our qPCR assay; pooling plasmids harboring the different *IDH1* mutants with *IDH1* WT DNA, we were able to detect at least 2% of mutated allele, except for the R132H mutant. Starting from mutated DNA extracted from FFPE samples and pooling with *IDH1* WT DNA, we were able to detect up to 5% of mutated alleles. The seriate dilutions of the R132G mutated sample revealed that qPCR could detect the mutation up to 0.3 ng of DNA. The highest sensitivity of qPCR compared to Sanger qPCR is uncontested. A possible strategy to improve our assay sensibility could be the use of a PNA clamp. This kind of probe is able to specifically block WT allele amplification in favor of mutant allele, if present. We did not use a PNA-clamp for our qPCR assay, because it was very important to avoid false negative results in plasma samples, due to inadequate sampling. Further, the WT allele amplification in each qPCR was a positive control. In recent years, other methods of mutation detection are frequently used, such as droplet PCR or NGS. Both the techniques are regarded as more sensitive compared to qPCR allowing the detection of less than 1% of mutated alleles, with the additional perk for NGS of multiple targets analyzed in the same experiment. NGS is frequently used in screening procedures, in particular to select patients with specific molecular alterations to be enrolled in clinical trials. However, the downside of such a high sensitivity is that the identification of very low amounts of mutated clones could be useless or even misleading from a clinical standpoint. Moreover, NGS is more prone to errors, due to the complexity of the technical procedures and to the interpretation of the large amount of data. Further, Gao and collaborators showed that NGS has certainly significant technical advantages, but qPCR showed similar analytical sensitivity and specificity, as well as high concordance with data obtained by NGS [21].

The innovation of our study is the capability of detecting the *IDH1*m in plasma samples, avoiding false negative results due to the absence of IDH1 copies in tested samples. In this case report, we were able to detect the mutation found in the FFPE sample also in cfDNA using the specific qPCR probe; by longitudinally analyzing the seriate plasma samples, we revealed a progressive increase of plasma concentration of *IDH1*m, which corresponded to the disease progression evidenced by the clinical examinations. Literature is conflicting about the significance of *IDH1*m for the biological aggressiveness and the course of the natural history of ICC. Hence, the prognostic role of *IDH1*m remains controversial in ICC. Against this backdrop, in our recent work, the comparison of primary and recurrent tumors revealed that in three cases the *IDH1*mR132x is detectable only in the recurrent tumors, suggesting for *IDH1*m a possible role in ICC progression after surgery [15]. Data from the present study add to these conclusions, supporting the hypothesis that plasma *IDH1*m could at least represent a biomarker of progression of advanced disease. The main limit of our study was that we tested the sensitivity of our qPCR assay analysis only in one patient, as a case report: further studies will be needed to validate our findings with a more conspicuous case series.

## 4. Materials and Methods

### 4.1. Patients

A retrospective series of 61 formalin-fixed, paraffin embedded (FFPE) samples, obtained from ICC patients diagnosed between 2005 and 2013, was collected from two Italian centers, the Institute of Candiolo and the Humanitas Clinical and Research Center of Rozzano. The Institute Review Board Ethical Committees of both centers approved the use of archival tissues for this specific research (Institute of Candiolo: 17 October 2018, ethic code 298/2018; Humanitas Clinical and Research Center: 18 October 2016, ethic code 1270). Three-micron thick slides were used for DNA extraction for each tumor. Tumor areas were identified by a pathologist by hematoxylin/eosin staining. Blood samples were collected from a single ICC patient, treated at our institute from May to September 2018. The patient was a 61 year old woman initially diagnosed with a stage IIIB, poorly differentiated (G3), multifocal cholangiocarcinoma, who had undergone a surgical intervention of right hepatectomy with locoregional lymphadenectomy. Upon disease recurrence, she was treated with three chemotherapy lines. At the time of disease recurrence, an assessment of somatic mutations was performed that revealed an *IDH1* R132C mutation. To test the efficiency of qPCR on plasma, blood samples were collected according to the Institute Review Board Ethical Committee. The patient signed the informed consent of PROFILING protocol (“Prospective study for the determination of molecular profiles of patients affected by tumors resistant to target therapies.”) Studio prospettico per la determinazione del profilo molecolare di resistenza alle terapie target in pazienti con malattie neoplastiche”, 001-IRCC-00IIS-10, version 6.1, FPO-IRCCS, l’Istituto di Ricovero e Cura a Carattere Scientifico Candiolo (TO), 23 June 2016).

Blood draws were performed at four time points—at the beginning of treatment for metastatic disease and then every 6 weeks during treatment, until disease progression, which occurred after 18 weeks of treatment.

### 4.2. DNA Extraction from FFPE Tissues

Genomic DNA was extracted using QIAamp DNA FFPE Mini kit (Qiagen, Hilden, Germany) following the manufacturer’s instructions. Briefly, tumor tissues were scraped and collected in 2 mL tubes. Tissues were then rehydrated by subsequent passages in xylene and ethanol; pellets were then treated and lyzed with proteinase K and lysis buffer over night at 56 °C. DNA purification and elution were performed using a column. DNA was quantified using the spectrophotometer Nanodrop 2000c (Thermo Fisher Scientific, Waltham, MA, USA).

### 4.3. DNA Isolation from Plasma Samples

For the liquid biopsy test, we started from plasma isolated from blood samples. Briefly, three EDTA tubes were centrifuged at 3000 rpm for 10 min at room temperature without brake, then supernatant was transferred in 15 mL polystyrene tubes and a second centrifugation was performed at 4000 rpm for 10 min at room temperature with brake. Plasma was then divided in aliquots and preserved at −80 °C. Cell free (cf) DNA was isolated using the MagMAX Cell-Free DNA Isolation Kit (Applied Biosystems, Foster City, CA, USA), following the manufacturer instructions. Lysis solution was mixed with 2 mL of plasma and incubated in agitation with cfDNA magnetic beads for 10 min. Beads were washed once with wash solution and twice with 80% ethanol. cfDNA was eluted with 35 μL of elution solution. Samples were stored at 4 °C until testing. All the cfDNA collected was subdivided into the test wells.

### 4.4. IDH1 Mutational Analysis

In order to analyze the mutational status of the hotspot codon 132 in the exon 4 of *IDH1* we used two different methods, the quantitative real-time allele-specific polymerase chain reaction (qPCR) system and the nested PCR followed by Sanger sequencing. The relative primers of each technique are indicated in Table S1.

The qPCR assay was performed on CFX96 (Bio-Rad, Hercules, CA, USA) using Takyon No Rox probe 5× MasterMix dTTP (Eurogentec SA, Seraing, Belgium). DNA samples isolated from FFPE tissues and plasma were amplified specific using primers, as shown in Table S1, flanking codon 132 (88 bp) at the final concentration of 200 nM. Probes were modified with locked nucleic acid (LNA) bases to enhance the assay specificity. Specific LNA-modified probes were synthesized for each mutation (R132C, R132L, R132G, R132H and R132S; FAM labeled) and wild-type allele (HEX labeled) (Eurogentec). In each reaction, the specific mutation and WT probes were mixed at different concentrations (final concentration 100 nM) as shown in Table S1.

As a positive control, a construct of 201 bp containing the sequence of *IDH1* exon 4 with the specific variation was inserted in a pUC57 plasmid (Eurogentec). Human female DNA reference (Agilent Technologies, Santa Clara, CA, USA) was used to test the WT probe.

Plasmids, probes and primers were quantified using a DS33 nano spectrophotometer (Denovix Inc., Wilmington, DE, USA) and Qubit dsDNA HS assay (Qiagen).

In order to test the assay sensitivity, plasmids and human DNA reference were diluted to build a four-point standard curve. Each specific plasmid (starting from 3 × 10^3^ to 3 copies) was tested in duplicate to increase the accuracy of the test.

The sensitivity of *IDH1* qPCR assay was analyzed in three different experiments: (1) pooling 3 × 10^3^ copies of WT *IDH1* DNA with different concentrations of plasmid harboring *IDH1* mutation (range 2–8%); (2) mixing DNA derived from 3 *IDH1* mutated samples (1 R132G; 2 R132L) with DNA from an *IDH1* wild-type sample (pure, 50% mutated and 10% mutated; 10 ng of total DNA); (3) diluting (1:10 for the first dilution, then 1:2) DNA derived from an R132G sample (from 200 to 0.3 ng).

Nested PCR was used to evaluate the mutational status of the hotspot codon 132 in the exon 4 of *IDH1*. The external and internal primers are indicated in Table S1. For the first round of PCR, the conditions were: 95 °C 5 min, 95 °C 30 s, 55 °C 30 s, 72 °C 45 s, repeated for 40 cycles with a final extension period of 72 °C 7 min. For the second round, a touch down PCR was used. PCR products (360 bp) were then purified using Wizard^®^ SV Gel and a PCR Clean-Up System (Promega, Italy) and sense and antisense sequences were obtained using forward and reverse internal primers, respectively. Each exon was sequenced using the BigDye Terminator Cycle sequence following the PE Applied Biosystem strategy and Applied Biosystem ABI PRISM3100 DNA Sequencer (Applied Biosystem). Mutations were confirmed performing two independent rounds of PCR amplifications and sequencing.

### 4.5. Analysis of IDH1 PCR Data

In order to evaluate the performance of each primer set, a standard curve was designed. A seriate dilution of the target (10-fold dilution for 4 log) was analyzed. We tested 5 plasmids each with a specific *IDH1* mutation and a genomic DNA as *IDH1* WT reference. Ct (threshold cycle) is the intersection between an amplification curve and a threshold line. It is a relative measure of the concentration of target in the PCR reaction. R^2^ is the coefficient of correlation obtained for the standard curve; its value should be >0.99 to consider the correlation as significant. Ideally, the efficiency (E) of a PCR should be around 100%, meaning that for each cycle the amount of product doubles. This efficiency is calculated from the slope(s) of the standard curves according to the following formula: E = (10^(−1/slope)^ − 1) × 100. For an efficiency of 100%, the slope is −3.32. A good reaction should have an efficiency between 90% and 110%, which corresponds to a slope between −3.58 and −3.10.

## 5. Conclusions

In conclusion, our qPCR assay may represent a useful, rapid and cheap tool to identify *IDH1* mutations in FFPE samples or to validate data obtained by high-throughput techniques. Of interest, this qPCR is able to detect the *IDH1* mutations also in plasma samples in cfDNA samples, avoiding false negative results. Hence, qPCR could reveal a suitable, non-invasive assay to integrate the standard clinical, radiologic and biohumoral restaging assessment in the course monitoring of *IDH1*m ICC.

## Figures and Tables

**Table 1:** Mean threshold cycle (Ct) obtained with different concentration of plasmids for the *IDH1*R132x mutations and wild-type (WT).

*IDH1* Status	3000 Copies (Ct)	300 Copies (Ct)	30 Copies (Ct)	3 Copies (Ct)	E	R^2^	Slope
WT	22.3	25.8	30.0	34.4	75.8	0.99	−4.1
R132C	27.3	30.6	34.0	37.1	98.9	0.99	−3.4
R132G	24.6	28.1	31.4	34.7	95.0	0.99	−3.5
R132H	28.2	30.8	33.1	35.8	133.6	0.99	−2.7
R132L	23.3	27.1	30.2	32.7	103.2	0.98	−3.3
R132S	22.6	26.4	29.7	32.6	102.9	0.99	−3.3

Ct: threshold cycle; E: efficiency; R^2^: coefficient of regression.

**Table 2:** Mean Ct obtained with different percentages of different *IDH1*R132x mutations inserted in plasmid and *IDH1* WT DNA.

*IDH1* Mutation	8% Mutated Plasmid		4% Mutated Plasmid		2% Mutated Plasmid	
	M-FAM Ct	WT-HEX Ct	M-FAM Ct	WT-HEX Ct	M-FAM Ct	WT-HEX Ct
R132C	26.0	28.9	26.9	28.6	27.9	28.5
R132G	27.0	28.9	27.8	28.5	28.9	28.5
R132H	ND	28.9	ND	28.6	ND	28.3
R132L	28.5	28.3	33.1	28.2	37.0	28.7
R132S	27.0	28.9	27.3	28.5	28.4	28.4

M-FAM: mutated *IDH1* labeled with FAM; WT-HEX: wild-type labeled HEX; ND: not detected.

**Table 3:** Mean Ct obtained with *IDH1* R132L and R132G mutated formalin-fixed paraffin-embedded (FFPE) samples pooled with *IDH1* WT DNA.

*IDH1* Mutated Sample		50%		10%	
ID	Mutation	M-FAM (Ct)	WT-HEX (Ct)	M-FAM (Ct)	WT-HEX (Ct)
#11	R132L	29.2	29.1	36.7	28.8
#41	R132L	27.6	27.7	ND	28.4
#61	R132G	27.8	29.1	31.3	29.0

M-FAM: mutated *IDH1* labeled with FAM; WT-HEX: wild-type labeled HEX; ND: not detected.

**Table 4:** *IDH1* hotspot mutations detected with the two techniques, respectively.

Patient ID	Mutation Detected with Sanger	Mutation Detected with qPCR
#11	R132L	R132L
#15		R132C
#21		R312C
#22	R132L	R132L
#41	R132L	R132L
#45		R312C
#46		R132L
#51	R132L	R132L
#57		R132L
#61	R132G	R132G

## Supplementary Materials

The following are available online at https://www.mdpi.com/2072-6694/11/4/454/s1, Figure S1: Ct values and amplification curves of qPCR of cfDNA obtained from 1 IDH1m patient analyzed at different time points. In blue the WT curves, in green the mutated ones, Figure S2: CT scan images at the baseline (A) and after 6 weeks (B). Scale bar represent 10 mm, Figure S3: CT scan images at 12 weeks (C) and 18 weeks (D) from baseline. Scale bar represent 10 mm, Figure S4: Sensitivity of nested PCR for the amplification of *IDH1* exon 4, Table S1: Primers used for IDH1 mutational analysis. Probes and oligonucleotides for qPCR.
