# Supplementary material for: Assessment of a High Sensitivity Method for Identification of IDH1 R132x Mutations in Tumors and Plasma of Intrahepatic Cholangiocarcinoma Patients
Source: Cancers (Basel). 2019 Mar 30;11(4):454. doi: 10.3390/cancers11040454 (PMC6521091; doi:10.3390/cancers11040454)
Supplement: Supplementary file 1 [file cancers-11-00454-s001.pdf]

Supplementary Materials: Assessment of a High Sensitivity Method for Identification of *IDH1* R132x Mutations in Tumors and Plasma of Intrahepatic Cholangiocarcinoma Patients

Caterina Peraldo-Neia, Maria Scatolini, Enrico Grosso, Pasquale Lombardi, Roberto Filippi, Chiara Raggi, Caterina Marchiò, Giuliana Cavalloni, Massimo Aglietta and Francesco Leone

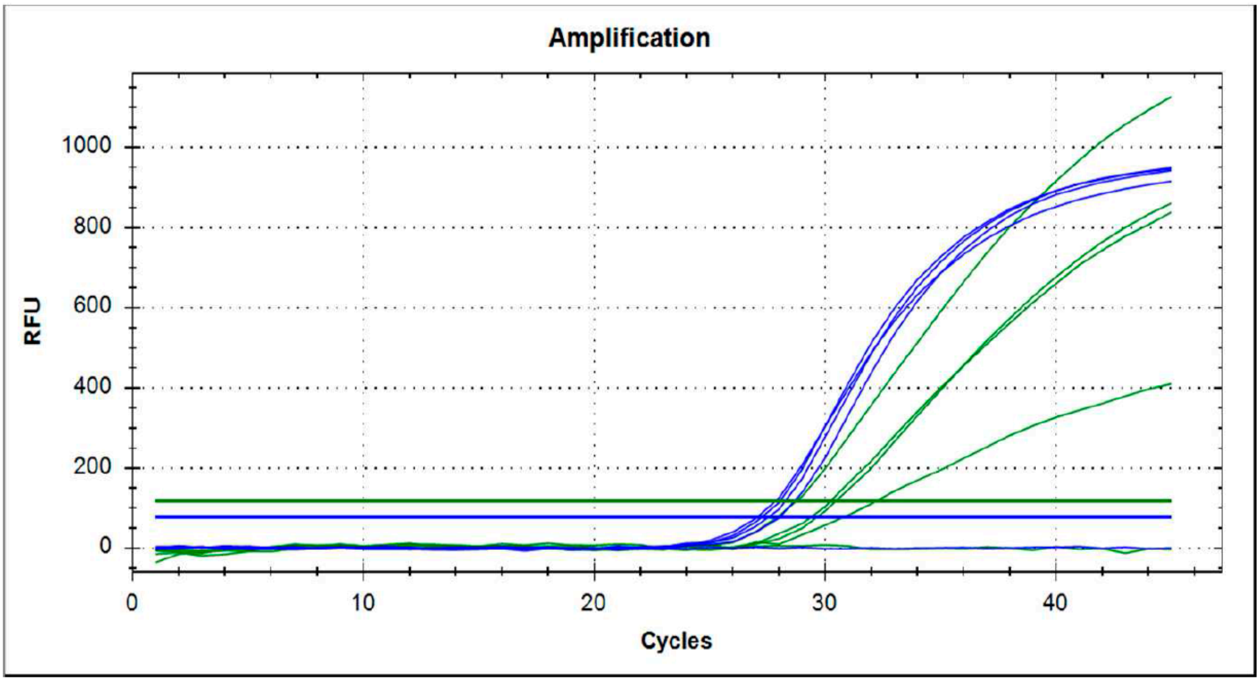

Figure S1. Ct values and amplification curves of qPCR of cfDNA obtained from 1 IDH1m patient analyzed at different time points. In blue the WT curves, in green the mutated ones.

A

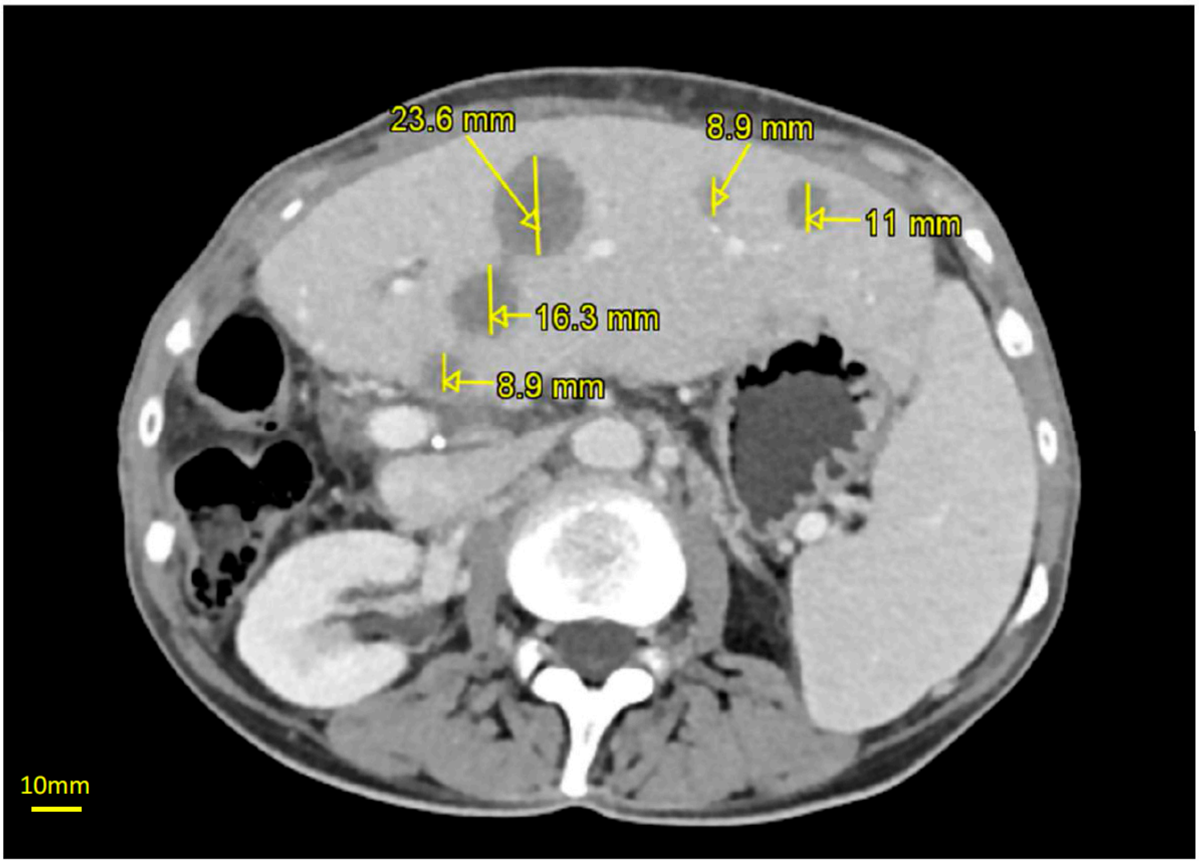

B

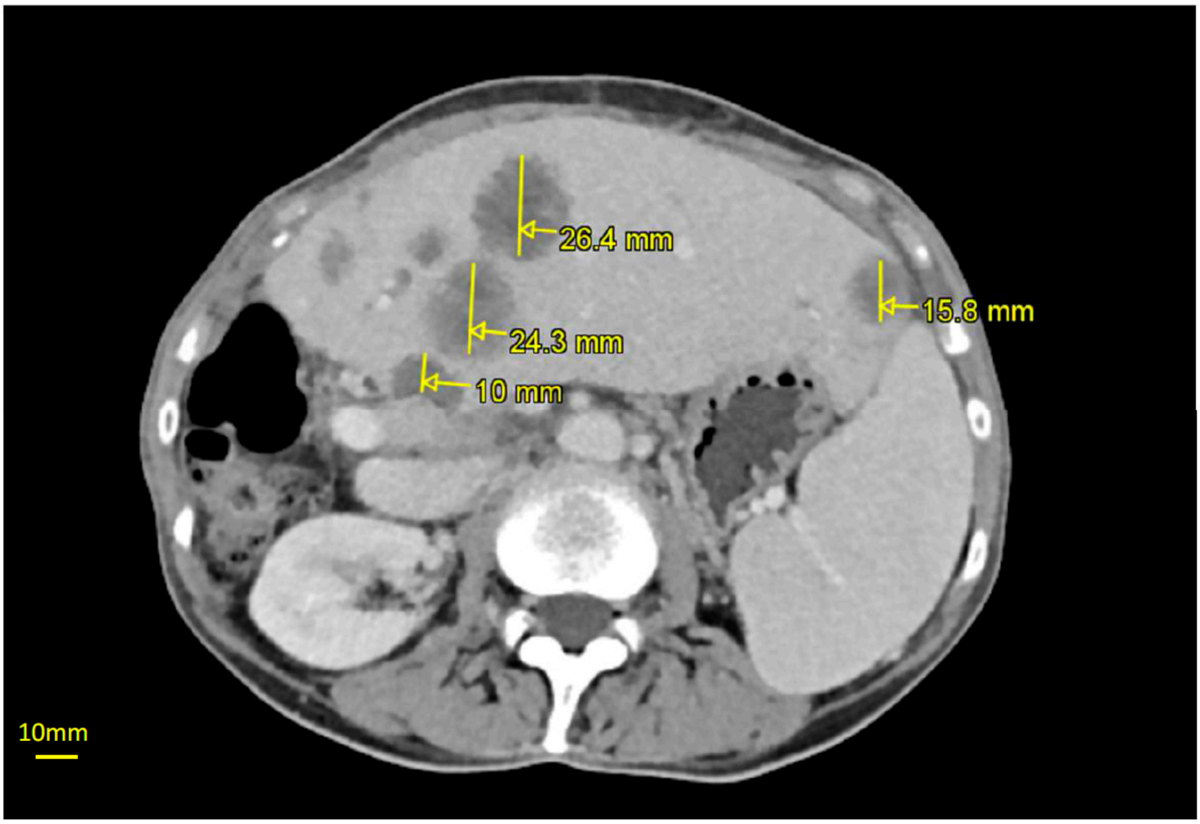

Figure S2. CT scan images at the baseline (**A**) and after 6 weeks (**B**). Scale bar represent 10 mm.

A

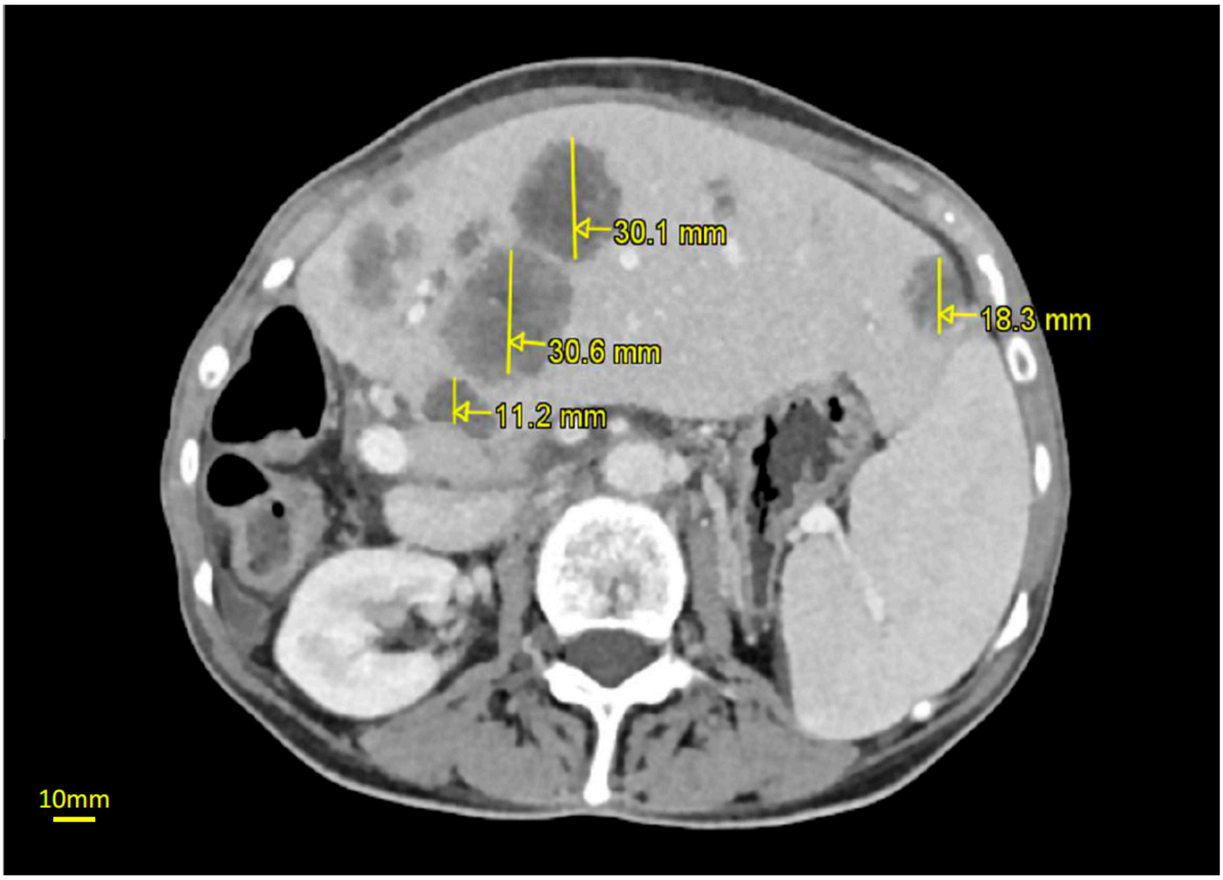

B

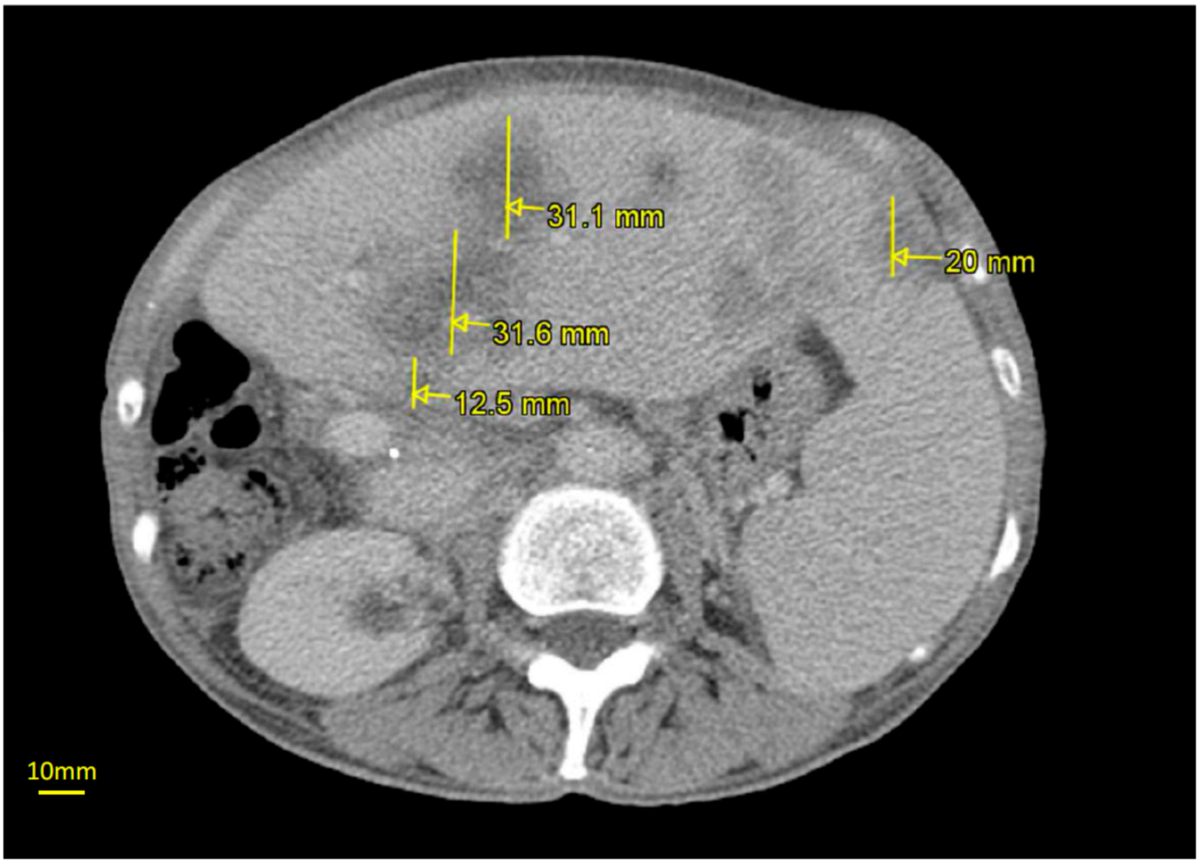

Figure S3. CT scan images at 12 weeks (**A**) and 18 weeks (**B**) from baseline. Scale bar represent 10 mm.

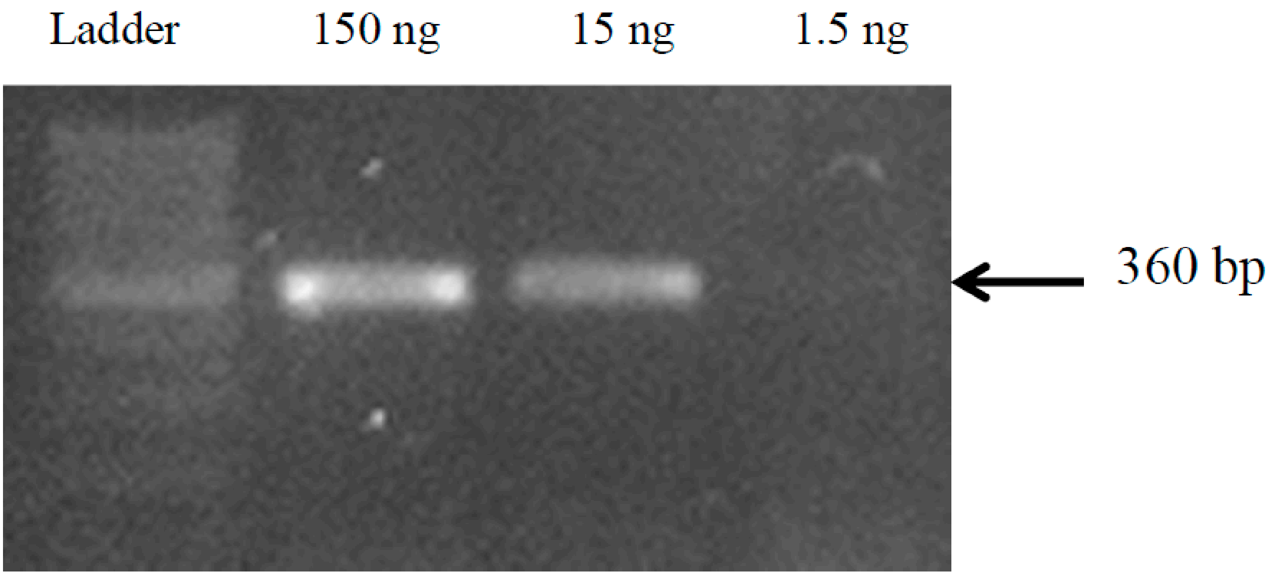

Figure S4. Sensitivity of nested PCR for the amplification of *IDH1* exon 4.
